# Preferential Homologous Chromosome Pairing in a Tetraploid Intergeneric Somatic Hybrid (*Citrus reticulata* + *Poncirus trifoliata*) Revealed by Molecular Marker Inheritance

**DOI:** 10.3389/fpls.2018.01557

**Published:** 2018-11-02

**Authors:** Mourad Kamiri, Marc Stift, Gilles Costantino, Dominique Dambier, Tariq Kabbage, Patrick Ollitrault, Yann Froelicher

**Affiliations:** ^1^UMR AGAP, CIRAD, San Giuliano, France; ^2^Ecology, Department of Biology, University of Konstanz, Konstanz, Germany; ^3^UMR AGAP, INRA, San Giuliano, France; ^4^UMR AGAP, CIRAD, Montpellier, France; ^5^Domaines Abbes Kabbage, Agadir, Morocco

**Keywords:** *Citrus*, somatic hybrid, tetraploid, disomic, tetrasomic, intermediate inheritance, SSR markers, SNP markers

## Abstract

The creation of intergeneric somatic hybrids between *Citrus* and *Poncirus* is an efficient approach for citrus rootstock breeding, offering the possibility of combining beneficial traits from both genera into novel rootstock lineages. These somatic hybrids are also used as parents for further tetraploid sexual breeding. In order to optimize these latter breeding schemes, it is essential to develop knowledge on the mode of inheritance in the intergeneric tetraploid hybrids. We assessed the meiotic behavior of an intergeneric tetraploid somatic hybrid resulting from symmetric protoplast fusion of diploid *Citrus reticulata* and diploid *Poncirus trifoliata*. The analysis was based on the segregation patterns of 16 SSR markers and 9 newly developed centromeric/pericentromeric SNP markers, representing all nine linkage groups of the *Citrus* genetic map. We found strong but incomplete preferential pairing between homologues of the same ancestral genome. The proportion of gametes that can be explained by random meiotic chromosome associations (τ) varied significantly between chromosomes, from 0.09 ± 0.02 to 0.47 ± 0.09, respectively, in chromosome 2 and 1. This intermediate inheritance between strict disomy and tetrasomy, with global preferential disomic tendency, resulted in a high level of intergeneric heterozygosity of the diploid gametes. Although limited, intergeneric recombinations occurred, whose observed rates, ranging from 0.09 to 0.29, respectively, in chromosome 2 and 1, were significantly correlated with τ. Such inheritance is of particular interest for rootstock breeding because a large part of the multi-trait value selected at the teraploid parent level is transmitted to the progeny, while the potential for some intergeneric recombination offers opportunities for generating plants with novel allelic combinations that can be targeted by selection.

## Introduction

Polyploidization is a major evolutionary pathway (Ramsey and Schemske, 2002; Adams and Wendel, 2005; Soltis et al., 2009) with important implications for breeding. In plants, polyploid lineages arise naturally through the union of unreduced gametes (Harlan and deWet, 1975; Ramsey and Schemske, 1998) and chromosome doubling of somatic cells (Aleza et al., 2011). They can also be artificially produced by treatment of cell tissue with colchicine (Aleza et al., 2009) or somatic hybridization (Grosser et al., 2000). Natural polyploidization is often associated with hybridization between species, resulting in allopolyploidy. Allopolyploids typically show strict preferential pairing in meiosis, resulting in disomic inheritance, without an opportunity for interspecific recombination (Soltis and Soltis, 2000; Pairon and Jacquemart, 2005). In allotetraploids, disomic inheritance leads diploid gametes to display interspecific heterozygosity throughout the genome. However, it has been recognized that even allopolyploids that combine strongly diverged parental genomes may have occasional nonhomologous chromosome pairing, leading to nonstrict disomic inheritance and intergenomic recombination (Stebbins, 1947; Sybenga, 1994, 1996; Udall and Wendel, 2006; Stift et al., 2008; Jeridi et al., 2012). Differential chromosome pairing affinities is therefore an essential component determining species evolution in natural polyploid populations. It is also a key parameter to design efficient polyploid breeding strategies, as it strongly impacts the inheritance of chromosome fragments and agronomical traits. Molecular marker analysis is a powerful approach to study the genetic structures of gametes produced by allopolyploid organisms, and in turn to estimate the preferential pairing pattern and its impacts on genome fragment inheritance and recombination (Allendorf and Danzmann, 1997; Barone et al., 2002; Jannoo et al., 2004; Bousalem et al., 2006; Landergott et al., 2006).

In citrus, polyploidy has been described to improve adaptation to different stresses and resilience (Mouhaya et al., 2010; Podda et al., 2013; Allario et al., 2013; Tan et al., 2015; Tan et al., 2017; Dutra de Souza et al., 2017; Oliveira et al., 2017; Oustric et al., 2017, 2018), and several teams worldwide have been developing rootstock breeding at the tetraploid level (Grosser and Chandler, 2000; Grosser et al., 2000, 2015; Ollitrault et al., 2000, 2007; Guo et al., 2007; Grosser and Gmitter, 2010; Dambier et al., 2011; Guerra et al., 2016). Many of these programs focus on combinations of *Citrus* species with *Poncirus*, a related genus. *Poncirus* and *Citrus* species are sexually compatible (Spiegel-Roy and Goldschmidt, 1996). However, molecular phylogenic studies based on whole genome resequencing data have demonstrated substantial genetic differentiation (Carbonell-Caballero et al., 2015; Wu et al., 2018). *Poncirus* has agriculturally useful traits such as cold adaptation, tolerances to *Phytophthora* species and nematodes, and resistance to the citrus tristeza virus (CTV; Yang et al., 2001). It has also been described to provide some tolerance to Huanglongbing, a devastating citrus disease caused by the phloem bacterium *Candidatus Liberibacter* sp. (Stover et al., 2010). However, *Poncirus* suffers iron chlorosis on alkaline soils and it is susceptible to salinity, which limits its use in some areas, particularly in the Mediterranean Basin (Spiegel-Roy and Goldschmidt, 1996). Rootstock breeding programs attempt to combine beneficial *Poncirus* traits with abiotic stress tolerance traits present in *Citrus* species. Although some interesting diploid intergeneric sexual hybrids [e.g., citrange (*P. trifoliata* × *C. sinensis*) and citrumelo (*P. trifoliata* × *C. paradisi*)] have been produced, the long generation times, partial apomixes, and segregation of beneficial allelic combinations due to the high heterozygosity of parental genotypes (Herrero et al., 1996; Ollitrault et al., 2003; Barkley et al., 2006; Wu et al., 2018) limit the efficiency of conventional diploid breeding. Somatic hybridization is an efficient alternative for citrus rootstock breeding as it allows breeders to combine favorable parental genes regardless of their heterozygosity level (Ollitrault et al., 1998; Grosser and Gmitter, 2010; Dambier et al., 2011; Ruiz et al., 2018). In citrus, somatic hybridization has also been successfully applied to diversify the tetraploid gene pool used as parent for triploid breeding (Grosser et al., 2000; Ollitrault et al., 2007, 2008) and to generate hybrids (Aleza et al., 2016b). The “Tetrazyg” approach (Grosser and Gmitter, 2010) was introduced to breed new tetraploid rootstock by sexual hybridization using selected allotetraploid somatic hybrid rootstock as parent material. In order to optimize the efficiency of the “Tetrazyg” strategy, it is essential to develop knowledge on the mode of inheritance in the allotetraploid hybrids.

Previous studies on tetraploid citrus revealed different meiotic behavior. Weak or no preferential pairing of homologous chromosomes (i.e., mostly tetrasomic inheritance) with bivalents and quadrivalent meiotic configurations was observed for a mandarin + lemon somatic hybrid (Kamiri et al., 2011), for a somatic hybrid between tangelo and pummelo (Xie et al., 2015), and doubled-diploid clementine (Aleza et al., 2016a). By contrast, the doubled-diploid “Mexican” lime had predominantly disomic segregation (Rouiss et al., 2018). However, no data were available on the inheritance mode of tetraploid *Citrus-Poncirus* intergeneric hybrids.

The aim of the present study was to investigate the mode of inheritance in a tetraploid intergeneric somatic hybrid called Flhorag1 obtained through protoplast fusion between *Citrus reticulata* cv “Willowleaf” mandarin and *Poncirus trifoliata* cv “Pomeroy” (Ollitrault et al., 2000). This somatic hybrid provided improved agronomic traits when used as rootstock with sweet orange (Dambier et al., 2011). For this purpose, a triploid progeny population (2*n* = 3*x* = 27) resulting from “Chandler” pummelo × Flhorag1 sexual hybridization was genotyped at 19 simple sequence repeat (SSR) and 9 single nucleotide polymorphism (SNP) loci to infer the allelic constitution of gametes produced by the somatic hybrid. The likelihood-based approaches proposed by Stift et al. (2008) for multi allelic loci and Aleza et al. (2016a) for di-allelic loci in duplex tetraploid were applied to analyze the meiotic behavior of Flhorag1. The implications for citrus rootstock breeding are discussed with a special focus on intergeneric heterozygosity restitution.

## Materials and Methods

### Plant Materials

An intergeneric somatic hybrid between a diploid (2*n* = 2*x* = 18) *Citrus reticulata* Blanco (“Willowleaf” mandarin SRA 133, hereafter called WLM) and a diploid (2*n* = 2*x* = 18) *Poncirus trifoliata* L. (“Pomeroy” trifoliate orange SRA 1074, hereafter called PON) was previously obtained by protoplast electrofusion (Ollitrault et al., 2000). To assess the inheritance of the tetraploid somatic hybrid (2*n* = 4*x* = 36; hereafter called Flhorag1), we performed a cross with diploid *Citrus maxima* (Burm.) Merr (2*n* = 2*x* = 18) (“Chandler” pummelo SRA 608, hereafter called CHA), using Flhorag1 as pollen donor. The pollen was collected on the mother tree of Flhorag1 (the plant directly regenerated from protoplast fusion).

CHA was chosen because it is self-incompatible, not apomictic, and it is genetically well differentiated from both WLM and PON. Cross was performed at the San Giuliano Research Station (Corsica, France). Recovered mature seeds were germinated *in vitro* in MT medium (Murashige and Tucker, 1969) supplemented with 30 g l^-1^ sucrose and 1 mg l^-1^ GA_3_ (Ollitrault et al., 1996). The obtained plants were grafted on “Volkamer” lemon (*C. limonia* Osbeck) and further grown in a growth chamber.

### Flow Cytometry and Cytogenetic Analyses

The ploidy of the progeny was determined by flow cytometry and confirmed by chromosome counts. For flow cytometry, approximately 0.5 cm^2^ of plantlet leaf and a similar amount of leaf tissue from a diploid reference *C. madurensis* (2*n* = 2*x* = 18) were chopped with a sharp razor blade in 250 μl of extraction buffer (Partec Cystain UV PreciseP) to isolate intact nuclei. After filtering the resulting suspension (30 μm pore size), 800 μl of DAPI (4-6-diamine-2-phenylindol) staining buffer was added (Partec, Cystain UV Precise P Staining Buffer). Samples were analyzed with a PA-I flow cytometer (Partec, Germany). We followed the protocol of D’Hont et al. (1996) for chromosome preparations. Briefly, fresh young leaf tissues were treated in 0.04% hydroxyquinoline for 4 h at room temperature and fixed for 48 h in 3:1 ethanol:acetic acid and stored at 4°C in 70% ethanol. The preparations were then treated for 20 min in 5N HCl and washed with distilled water. Finally, the tissue was deposited on microscope slides, stained with a drop of DAPI staining buffer (Partec, Cystain UV Precise P Staining Buffer), and squashed. Chromosomes were counted under an Eclipse 80i fluorescence microscope (Nikon Instruments, France).

### DNA Extraction

DNA was extracted from leaves using a modified mixed alkyl trimethyl ammonium bromide (MATAB) procedure (Gawel and Jarret, 1991). The DNA concentration was determined using the Hoechst 33258 (Sigma Chemical Co., MO, United States) protocol (Sambrook and Russell, 2006). Samples were diluted with MQ sterile water and stored at -20°C until use.

### SSR Amplification

From previously identified, characterized, and mapped SSR loci (Froelicher et al., 2008; Luro et al., 2008; Ollitrault et al., 2010, 2012), we selected 19 loci (Table 1) that (i) were polymorphic between WLM and PON (diploid parents of the tetraploid somatic hybrid) and the seed parent CHA, and (ii) represented each of the nine linkage groups of the clementine genetic map (Ollitrault et al., 2012). Primers were labeled with WELLRED fluorochrome PA-2(dye2), PA-3(dye3), or PA-4(dye4) (Beckman-Coulter, CA, United States) and synthesized by Sigma-Aldrich (France). Among the selected markers, the mCrCIR02F12 locus was known to have a frequent null allele in *Poncirus* accessions (unpublished data), and the PON parent was suspected to be heterozygous (A0).

**Table 1:** Characteristics of selected SSR markers used for the Willowleaf mandarin + Pomeroy *Poncirus* somatic hybrid (Flhorag1) allelic inheritance.

Locus	EMBL	Linkage Group	Primer Sequence (5′–3′)	Tm (C°)
CiBE5055	ET111355	1	F:AACAGTGGTTCTGGGAAATAG	55
			R:GGTGGTCTCAAAGTCATCATC	
MEST431	FC883898	1	F: GAGCTCAAAACAATAGCCGC	55
			R: CATACCTCCCCGTCCATCTA	
mCrCIR02D09	FR677569	2	F:AATGATGAGGGTAAAGATG	55
			R:ACCCATCACAAAACAGA	
MEST46	FC901824	2	F: AACCAGAATCAGAACCCGA	55
			R: GGTGAGCATCTGGACGACTT	
mCrCIR02G12	FR677575	3	F:AAACCGAAATACAAGAGTG	55
			R:TCCACAAACAATACAACG	
mCrCIR02D04b	FR677564	4	F:CTCTCTTTCCCCATTAGA	50
			R:AGCAAACCCCACAAC	
mCrCIR03D12a	FR677577	4	F:GCCATAAGCCCTTTCT	50
			R:CCCACAACCATCACC	
mCrCIR07D06	FR677581	4	F:CCTTTTCACAGTTTGCTAT	55
			R:TCAATTCCTCTAGTGTGTGT	
mCrCIR03F05	FR692364	4	F:CTAAGGAAGAGTAGAGAGCA	50
			R:TAAAATCCAAGGTTCCA	
mCrCIR01F08a	AM489737	5	F:ATGAGCTAAAGAGAAGAGG	50
			R:GGACTCAACACAACACAA	
mCrCIR07E12	AM489750	5	F:TGTAGTCAAAAGCATCAC	50
			R:TCTATGATTCCTGACTTTA	
mCrCIR02F12	FR677570	6	F:GGCCATTTCTCTGATG	55
			R:TAACTGAGGGATTGGTTT	
mCrCIR02D03	FR692360	7	F:CAGACAACAGAAAACCAA	55
			R:GACCATTTTCCACTCAA	
mCrCIR07E05	AM489749	7	F:GGAGAACAAAACACAATG	50
			R:ATCTTTCGGACAATCTT	
mCrCIR02A09	FR677568	8	F:ACAGAAGGTAGTATTTTAGGG	50
			R:TTGTTTGGATGGGAAG	
mCrCIR07B05	AM489747	8	F:TTTGTTCTTTTTGGTCTTTT	50
			R:CTTTTCTTTCCTAGTTTCCC	
mCrCIR02G02	FR677572	8	F:CAATAAGAAAACGCAGG	55
			R:TGGTAGAGAAACAGAGGTG	
mCrCIR07C09	AJ567410	9	F:GACCCTGCCTCCAAAGTATC	55
			R:GTGGCTGTTGAGGGGTTG	
mCrCIR02B07	AJ567403	9	F:CAGCTCAACATGAAAGG	50
			R:TTGGAGAACAGGATGG	

*EMBL, accession number in the EMBL–EBI database (https://www.ebi.ac.uk/).*

PCR reactions were performed in 20 μl with 1× Taq buffer, 1.5 mM MgCl_2_, 0.8 U Taq DNA polymerase, 0.5 ng/μl template DNA, 0.2 mM dNTPs, 0.4 μM forward primer, and 0.4 μM reverse primer in an AG Primus 96 plus thermocycler (MWG, Germany). The PCR program consisted of 5 min initial denaturation (94°C), followed by 40 cycles of 30 s denaturation (94°C), 1 min primer annealing (50 or 55°C depending on the primers, Table 1), and 45 s extension (72°C), and a final extension of 4 min (72°C). Fragment analysis and allele calling were done by capillary electrophoresis using the CEQ 8800 genetic analyzer and software (Beckman Coulter, CA, United States). As CHA is totally differentiated from the WLM + PON tetraploid parent, the diploid gamete genotypes were directly inferred from the triploid hybrid genotypes by removing the specific CHA alleles.

### Centromeric SNP Development and Analysis

We have developed new SNP markers in centromeric region of the nine citrus chromosomes. “Pomeroy” trifoliate, “Willow leaf” mandarin and “Chandler” pummelo whole genome resequencing data (available, respectively, with SRX2442480, SRX372685, and SRX372688 SRA number in NCBI database) were mapped on the haploid clementine reference genome (Wu et al., 2014) using “BWA-MEM, v0.7.12-r1039” (Li and Durbin, 2010) and variant calling was performed with GATK (McKenna et al., 2010). To be able to infer the intergeneric gamete structure from the genotyping of the triploid issued from diploid Chandler diploid X (Willow Leaf + Pomeroy) tetraploid hybridizations, we selected SNPs homozygous for “Willow leaf” (AA), “Chandler” (AA), and “Pomeroy” (BB) in gene sequences of the identified centromeric/pericentromeric regions (Wu et al., 2014; Aleza et al., 2015). The genetic distances to the centromeres were inferred from available genetic mapping data (Ollitrault et al., 2012; Aleza et al., 2015) considering the flanking markers in the physical sequence (Wu et al., 2014). We were able to select efficient markers located at less than 1 cM for seven chromosomes, at less than 2 cM for chromosome 5 and less than 5cM for chromosome 8 (Table 2). The selected SNPs were analyzed with KASPar^TM^ Genotyping System (a competitive, allele-specific dual Förster resonance energy transfer–based assay). Primers were designed by LGC Genomics from the SNP locus flanking sequence (Supplementary Material 1). Detailed explanation on the specific conditions and reagents used can be found in Cuppen (2007). Identification of allele dosages in heterozygous triploid hybrids was carried out on the basis of relative allele signals, as described by Cuenca et al. (2013). For the nine SNP markers, the allelic configuration was as follow: “Chandler” (AA) × ”Flhorag1” (AABB) producing AAA, AAB, and ABB triploid progenies with direct inference of the “Flhorag1” diploid gametes (AA, AB, and BB, respectively).

**Table 2:** Location of nine centromeric/pericentromeric SNP loci fully distinguishing “Pomeroy” poncirus from “willow leaf” mandarin and “Chandler” pummel.

Marker	Scaffold	Position	SNP	Gene ID	D/Cent (cM)
P1_16582061	1	16582061	[C/T]	Ciclev10007229m.g	<1
P2_19903193	2	19903193	[T/C]	Ciclev10014147m.g	<1
P3_16287238	3	16287238	[T/C]	Ciclev10024301m.g	<1
P4_9505439	4	9505439	[A/G]	Ciclev10031824m.g	<1
P5_20142568	5	20142568	[T/A]	Ciclev10000575m.g	<2
P6_3130249	6	3130249	[G/A]	Ciclev10011214m.g	<1
P7_15458045	7	15458045	[A/G]	Ciclev10025280m.g	<1
P8_16631925	8	16631925	[A/T]	Ciclev10030352m.g	<5
P9_12062066	9	12062066	[C/T]	Ciclev10006749m.g	<1

*SNP positions, gene ID, and flanking position came from the haploid clementine reference sequence (Wu et al., 2014; https://phytozome.jgi.doe.gov/pz/portal.html#!info?alias=Org_Cclementina).*

### Data Analysis

Pluri-allelic SSR markers: first we tested if the SSR allele frequencies observed in gametes deviated from those expected based on the parental genotypes (chi-square goodness-of-fit test), which would indicate the presence of null alleles or selection against particular alleles. Subsequently, we applied a likelihood based approach that simultaneously estimates two parameters: (1) τ – the proportion of gamete allelic constitutions that can be explained by random tetrasomic segregation and (2) β – the double reduction frequency relative to τ (Stift et al., 2008). This was done for each preferential pairing scenario (e.g., for a marker displaying the ABCD genotype for WLM + PON, we evaluated three pairing scenarios: A with B, C with D; A with C, B with D; A with D, B with C). For parameter estimation, we used the constrained non-linear regression (CNLR) function implemented in SPSS 15.0 (SPSS syntax file^1^). We then used a likelihood ratio test (LRT) evaluated against a compound distribution of $½\chi_{0}^{2}+½\chi_{1}^{2}$ (Self and Liang, 1987) to determine if the model with the estimated τ explained the data significantly better than a random null model (i.e., a model with strict tetrasomic segregation, τ = 1). For loci for which “Flhorag1” had less than four alleles, we considered the possibility of null alleles using a G-test for contingency tables to evaluate the most probable constitution. In case of three alleles, we compared abc0 and abcc (for parents of the somatic hybrid genotyped ab and cc), and in the case of two allele aabb, aab0, abb0, or ab00 (parents of the somatic hybrid aa and bb).

Di-allelic centromeric/pericentromeric SNP markers: for centromeric diallelic markers the segregation model for duplex tetraploid parents (AABB configuration) is greatly simplified because the double reduction effect can be missed and the segregation pattern is a direct function of τ. Therefore, for these markers τ was estimated by the maximum likelihood approach proposed by Aleza et al. (2016a).

Genetic dissimilarities between diploid gametes were estimated with the DARwin 6.0 software (Perrier and Jacquemoud-Collet, 2006) using the “simple matching” dissimilarity index using the pluriallelic SSRs markers to integrate intergeneric and intraspecific polymorphisms.

## Results

### Ploidy Level Determination

The cross between the diploid maternal parent (CHA) and the tetraploid somatic hybrid (“Flhorag1”) resulted in 63 fully developed seeds collected from 41 fruits, from which 59 plantlets germinated. Flow cytometry indicated that the plantlets were triploid, with one tetraploid exception (data not shown). Chromosome counts (Supplementary Material 2) confirmed the flow cytometry results. The segregation analyses were performed for the 58 triploid hybrids.

### SSR Genotyping

For 15 out of the 19 SSRs markers, allele frequencies observed in the gametes did not significantly differ from expected frequencies based on the “Flhorag1” genotype, that is, there was no segregation distortion. This confirmed the correct determination of allele doses in the somatic hybrid and the absence of null alleles. For one marker (mCrCIR02F12), a scenario assuming the previously suspected presence of a null allele in the PON genome could explain the observed allele frequencies. Moreover, the “microsatellite DNA allele counting—peak ratios” (MAC-PR) method based on relative areas of the peaks of the different alleles provided additional evidence of a null allele at this locus.

At the three remaining loci (mCrCIR02D03, mCrCIR07C09, and mCrCIR03F05), the progeny allele frequencies were significantly distorted (*P* < 0.05) and remained so when scenarios were considered involving null alleles in either WLM or PON (Table 3). In addition, MAC-PR provided no evidence for null alleles at these loci. They were excluded from further analyses, so the segregation analyses were performed with 16 loci with Mendelian segregation. For these 16 SSR markers, the MAC-PR analysis of “Flhorag1” confirmed a full addition of parental (WLM and PON) alleles.

**Table 3:** Distribution of inferred diploid gamete genotypes that produced CHA × (WLM + PON) progeny at the 19 studied loci.

Locus	Linkage Group	Parental Genotypes		Inferred Genotypes of Diploid Gametes														Allelic Distortion *p* Value
		WLM	PON							
**Segregation with two or three different alleles**				**AA**		**AB**		**AC**		**BB**		**CC**		**BC**				

CiBE5055	1	BC	AA	5		22		28		0		0		3				0.68
MEST431	1	BB	AA	5		46		n.a.		7		n.a.		n.a.				0.68
mCrCIR02D09	2	AB	CC	1		0		25		1		1		30				0.79
MEST46	2	BB	AA	2		56		n.a.		0		n.a.		n.a.				0.71
mCrCIR02G12	3	CC	AB	2		3		25		1		0		27				0.54
mCrCIR02D04b	4	AA	BC	5		28		24		0		0		1				0.66
mCrCIR03D12a	4	AA	BC	1		28		24		1		1		3				0.66
mCrCIR07D06	4	AB	CC	0		0		26		0		6		26				0.54
mCrCIR03F05	4	BB	AC	2		27		8		0		4		17				0.02^∗^
mCrCIR01F08a	5	BB	AA	3		50		n.a		5		n.a		n.a				0.71
mCrCIR07E12	5	AC	BB	0		26		5		2		0		25				0.79
mCrCIR02D03	7	AB	AC	1		5		28		1		1		22				1.14E-06^∗^
mCrCIR07E05	7	BB	AA	4		51		n.a.		3		n.a.		n.a.				0.85
mCrCIR02A09	8	CC	AB	1		1		28		0		2		26				0.87
mCrCIR07B05	8	BB	AA	1		55		n.a.		2		n.a.		n.a.				0.85
mCrCIR07C09	9	AC	BC	1		20		7		1		11		18				0.04^∗^
mCrCIR02B07	9	AC	BB	0		25		3		5		2		23				0.97
**Segregation with four different alleles**				**AA**	**AB**		**AC**	**AD**	**BB**	**BC**		**BD**	**CC**	**CD**		**DD**		
mCrCIR02G02	8	AB	CD	0	2		23	7	0	13		12	0	1		0		0.14
**Segregation including a null allele**				**AA**	**AB**		**AC**	**A0**	**BB**	**BC**		**B0**	**CC**	**C0**		**00**		
mCrCIR02F12	6	AB	C0	0	3		14	10	0		21	9	0		1		0	0.16

*WLM, *Citrus reticulata* cv Willowleaf mandarin; PON, *Poncirus trifoliata* cv Pomeroy; Flhorag1, *Citrus reticulata* cv Willowleaf mandarin + *Poncirus trifoliata* cv Pomeroy; A. B. C. D, allele length (A represents the longest allele); 0, null allele. For three of these loci, allelic distortion was observed (^∗^). Corresponding P values for allelic distortion are given.*

### SSR Inheritance in the Flhorag1 Tetraploid Somatic Hybrid

**FIGURE 1:**
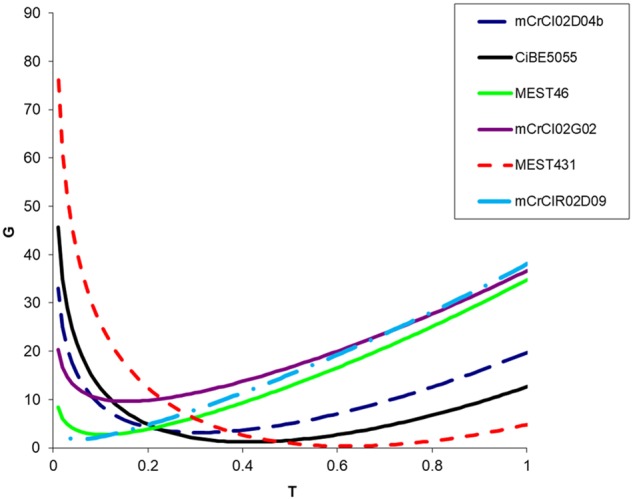
Example of deviance (G) for six observed SSR loci segregations in the “Flhorag1” somatic hybrid gametes with inheritance models ranging from τ = 0 (full disomic) to τ = 1 (full tetrasomic). Among the 16 analyzed SSRs markers Mest431 and mCrCIR02D09 display, respectively, the higher (0.58) and lower (0.07) τ values.

For all 16 analyzed loci, the estimated value of τ (proportion of gametes that can be explained by random meiotic chromosome associations) ranged from 0.07 to 0.58 (Figure 1 and Table 4). For each marker, fits between full tetrasomic inheritance model (τ = 1) and the best fitting intermediate model were compared. The LRT values were all highly significant, with *p* values ranging from 7.56E-09 to 1.78E-02 (Table 4). Preferential pairing was always between homologous chromosomes (between chromosomes derived from the same parental genome; i.e., mandarin or *Poncirus*).

**Table 4:** Fitting the inheritance model and intraparental and interparental heterozygosity rates on the segregation of 16 SSR loci in the “Flhorag1” diploid gametes.

SSR	LG	WLM	PON	Pref Pairing	Best model				% Heterozygosity	
					β	*τ*	LRT	*p* value	Intra-specific	Inter-generic
CiBE5055	1	BC	AA	BC/AA	–	0.41	5.79	8.04E-03	5%	86%
MEST431	1	AA	BB	AA/BB	–	0.58	4.41	1.78E-02	0%	79%
mCrCIR02D09	2	AB	CC	AB/CC	0.07	0.07	20.03	4.00E-06	2%	95%
MEST46	2	BB	AA	AA/BB	–	0.1	32.04	7.56E-09	0%	97%
mCrCIR02G12	3	CC	AB	CC/AB	0.05	0.21	12.74	1.79E-04	5%	90%
mCrCIR02D04b	4	AA	BC	AA/BC	–	0.31	8.39	1.89E-03	2%	90%
mCrCIR03D12a	4	AA	BC	AA/BC	0.04	0.23	11.42	3.64E-04	5%	90%
mCrCIR07D06	4	AB	CC	AB/CC	–	0.31	8.39	1.89E-03	0%	90%
mCrCIR01F08a	5	BB	AA	AA/BB	–	0.41	11.45	3.57E-04	0%	86%
mCrCIR07E12	5	AC	BB	AC/BB	–	0.36	7.01	4.06E-03	9%	88%
mCrCIR02F12	6	AB	C0	AB/C0	–	0.21	11.73	3.07E-04	7%	93%
mCrCIR07E05	7	BB	AA	AA/BB	–	0.36	13.86	9.90E-05	0%	88%
mCrCIR02A09	8	CC	AB	CC/AB	0.03	0.14	14.19	8.20E-05	2%	93%
mCrCIR07B05	8	BB	AA	AA/BB	–	0.16	27.26	8.88E-08	0%	95%
mCrCIR02G02	8	AB	CD	AB/CD	–	0.16	13.79	1.02E-04	5%	95%
mCrCIR02B07	9	AC	BB	AC/BB	0.07	0.38	6.34	5.89E-03	5%	83%

*Comparisons of fit were performed between the tetrasomic null model and the best fitting intermediate model. LRT values were evaluated as described in the Materials and methods and were significant at the indicated level. WLM, *Citrus reticulata* cv Willowleaf mandarin; PON, *Poncirus trifoliata cv Pomeroy*; A. B. C. D, allele length (A represents the longest allele); 0, null allele.*

The double reduction rate could only be estimated at 10 loci. Indeed, for MEST046, MEST431, mCrCIR01F08a, mCrCIR07B05, and mCrCIR07E05 markers, WLM and PON were both homozygous with a double homoduplex conformation (AA, BB). For the mCrCIR02F12 locus, null alleles prevented double reduction gamete identification. Double reduction was detected for five loci (mCrCIR02D09, mCrCIR02G12, mCrCIR03D12a, mCrCIR02A09, and mCrCIR 02B07) with β estimates ranging from 0.03 to 0.07 (Table 4).

### SNP Inheritance in the Flhorag1 Tetraploid Somatic Hybrid

For each SNP locus, “Flhorag1” was heterozygous with equilibrated allelic doses (AABB). No allelic distortion was observed for the nine diallelic SNPs. They revealed similar intergeneric heterozygosity restitution and τ values than the ones estimated on the same LGs with SSRs (Table 5). Intergeneric heterozygosity varied between 86% in LGs 1 and 9 to 97% in LG 2. In accordance, the lower τ value (0.11) was inferred for LG2 and the higher (0.42) for LG1 and LG9.

**Table 5:** Fitting the inheritance model and intergeneric heterozygosity rates on the segregation of nine centromeric SNP loci in the “Flhorag1” diploid gametes.

	Parental Genotypes				Diploid Gametes			Allelic Distortion *p* value	*τ*	Intergeneric Heterozygosity
					Genotypes					
	LG	WLM	PON	Flhorag1	AA	AB	BB		
P1_16582061	1	AA	BB	AABB	5	50	3	0.71	0.42	86%
P2_19903193	2	AA	BB	AABB	1	56	1	1.00	0.11	97%
P3_16287238	3	AA	BB	AABB	3	54	1	0.71	0.21	93%
P4_9505439	4	AA	BB	AABB	4	52	2	0.71	0.31	90%
P5_20142568	5	AA	BB	AABB	2	52	4	0.71	0.31	90%
P6_3130249	6	AA	BB	AABB	2	55	1	0.85	0.16	95%
P7_15458045	7	AA	BB	AABB	2	52	4	0.71	0.31	90%
P8_16631925	8	AA	BB	AABB	1	55	2	0.85	0.16	95%
P9_12062066	9	AA	BB	AABB	5	50	3	0.71	0.42	86%

### Distribution of τ Variations of SSR and SNP Loci Among the Different LGs

Loci belonging to the same linkage group (LG) tended to have similar τ values with significant differences between LGs. LG1 (CiBE5055, MEST431, P1_16582061; τ = 0.47 ± 0.09); LG2 (mCrCIR02D09, MEST046, P2_19903193; τ = 0.09 ± 0.02); LG3 (mCrCIR02G12, P3_16287238; τ = 0.21 ± 0.00); LG4 (mCrCIR02D04b, mCrCIR03D12a, mCrCIR07D06, P4_9505439; τ = 0.29 ± 0.03); LG5 (mCrCIR01F08a, mCrCIR07E12, P5_20142568; τ = 0.36 ± 0.05); LG6 (mCrCIR02F12; P6_3130249; τ = 0.18 ± 0.04); LG7 (mCrCIR07E05; P7_15458045; τ = 0.34 ± 0.03); LG8 (mCrCIR02A09, mCrCIR07B05, mCrCIR02G02, P8_16631925; τ = 0.15 ± 0.01); LG9 (mCrCIR02B07, P9_12062066; τ = 0.40 ± 0.02. Therefore, we can consider that LG1 has an intermediary inheritance while the other LGs had a preferential disomic tendency highly marked for LG2 and LG8.

### Heterozygosity Transmission and Gamete Diversity

**FIGURE 2:**
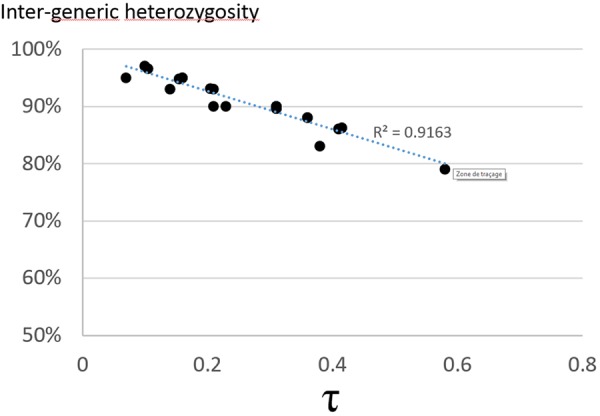
Correlation between the intergeneric heterozygosity transmission rate and the τ value for the 16 SSR and 9 SNP markers.

**FIGURE 3:**
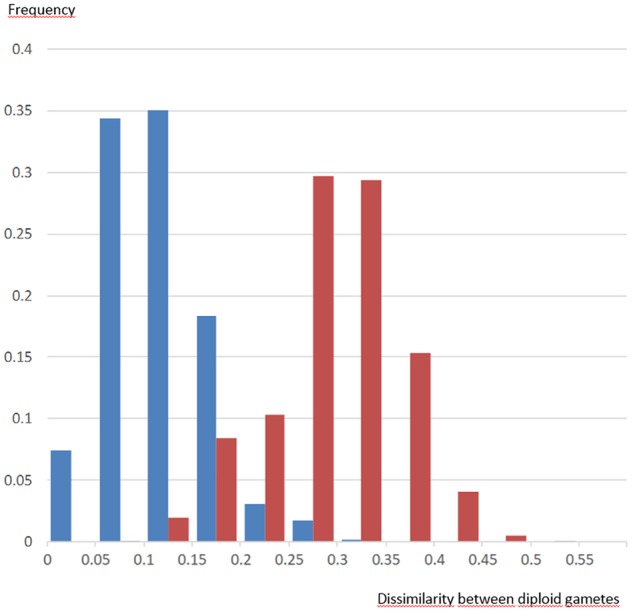
Distribution of genetic dissimilarities between diploid gametes of the Flhorag1 somatic hybrid based on intergeneric segregation (blue) and all allele segregation (red).

Diploid gametes from this tetraploid intergeneric somatic hybrid transmitted both intraspecific and intergeneric heterozygosity. The intergeneric heterozygosity transmission rate was directly linked with τ, and decreased linearly from 97 to 79% with increasing τ values (Figure 2). When analyzing the multilocus gametic structure over the 25 loci with complete allelic differentiation between *Citrus* and *Poncirus*, we found five diploid gametes (among the 58 analyzed) with complete intergeneric heterozygosity (Supplementary Material 3). The average intergeneric and intrageneric heterozygosity transmission were, respectively, 90.3 and 4.1%. Intergeneric recombinations were detected from multiloci pattern within each LG (Supplementary Material 3), whose frequencies (0.29, 0.09, 0.14, 0.24, 0.26, 0.10, 0.16, 0.14, 0.26 in LGs 1 to 9, respectively) were significantly related (*R*^2^ = 0.82) with the τ average values by LGs. We compared the distribution of dissimilarities between gametes considering all SSR alleles (intraspecific and intergeneric diversity) or solely the intergeneric origin of alleles (i.e., only two alleles considered: a poncirus allele P and a mandarin allele M and therefore segregation between PP, MM, and PM genotypes). Preferential pairing and consecutive predominant intergeneric heterozygosity transmission resulted in a relatively low intergeneric diversity contribution and gamete genetic diversity seemed to be mainly due to parental intraspecific diversity segregation (Figure 3).

## Discussion

### Intermediate Inheritance With a Preferential Disomic Trend Detected in the *C. reticulata* + *P. trifoliata* (Flhorag1) Intergeneric Tetraploid Somatic Hybrid

All 16 SSR and 9 SNP markers studied displayed intermediate inheritance with a preferential disomic tendency. The gamete proportions explained by random meiotic chromosome association (τ) ranged from 7 to 58%, and for all markers preferential pairing occurred between chromosomes derived from the same parent (i.e., WLM or PON). Such intermediate inheritance with a disomic tendency was also described for a doubled-diploid “Mexican lime” (Rouiss et al., 2018) belonging to *C. x aurantiifolia* (Christm.) Swingle, a species of interspecific origin (*C. micrantha* Wester *x C. medica* L.; Nicolosi et al., 2000; Curk et al., 2016; Wu et al., 2018). The levels of preferential pairing revealed for “Flhorag1” and the doubled-diploid “Mexican lime” are much higher than that observed for (i) an interspecific somatic hybrid (*C. reticulata + C. lemon*) within the *Citrus* genus, where random tetrasomic inheritance (τ = 1) could only be rejected for 8 out of 17 markers, with τ ranging from 0.24 to 0.95 (Kamiri, et al. 2011), and (ii) a doubled-diploid clementine displaying tetrasomic segregation for five chromosomes, intermediate segregation with a tetrasomic tendency for three chromosomes and intermediate segregation with a disomic tendency for only one chromosome (Aleza et al., 2016a). Preferential pairing variations between tetraploid citrus reveal different levels of divergence between their constitutive genomes.

Cytogenetic study of meiosis behavior is another approach to analyze chromosome affinity in polyploids. Indeed, tetravalent formation testifies for chromosome homology between parents while they should be precluded in wide allotetraploids (assuming the absence of large structural variation). Few cytogenetic studies were performed in tetraploid somatic hybrids and most concerned interspecific hybrids within the *Citrus* genus. Del Bosco et al. (1999) were the first to report the microsporogenesis in a Citrus interspecific tetraploid somatic hybrid between “Valencia” sweet orange and “Femminello” lemon. They observed frequent tetravalents. One of these tetravalents by meiotic cell was related to a reciprocal translocation in sweet-orange but the additional tetravalents were considered as a consequence of intergenomic pairing. Similar observation for frequent tetravalents were reported for “Hamlin” sweet orange + “Rough” Lemon and “Key” lime + “Valencia” sweet orange (Chen et al., 2004) and “Willow leaf” mandarin + “Eureka” lemon (Kamiri et al., 2011). For the last somatic hybrid, this cytogenetic observation was associated with preferential tetrasomic inheritance of molecular markers. However, as previously mentioned, reciprocal or inverted translocations can result in tetravalent formation even in diploid parents as observed in “Valencia” sweet orange (Del Bosco et al., 1999) or Mexican lime (Rouiss et al., 2018). Therefore, cytogenetic observations should be associated with marker segregation analysis for a full understanding of tetraploid meiosis behavior. In the case of mandarin + poncirus somatic hybrids, the predominant preferential pairing between homologous chromosomes inferred in “Flhorag1” from molecular marker segregation data is in agreement with the previous cytogenetic study conducted in an intergeneric citrus somatic hybrid between “Cleopatra” mandarin and “Argentine” poncirus (unpublished results mentioned in Chen et al., 2004). Indeed, they observed a high percentage of bivalents, suggesting low chromosome homology between the fusion parents.

Recent whole nuclear genome resequencing data revealed an important differentiation of *Poncirus trifoliata* from the *Citrus* species clade (Wu et al., 2018). This genomic differentiation level is in line with the preferential disomic inheritance observed for our *C. reticulata* + *P. trifoliata* intergeneric somatic hybrid. Interestingly, our finding that preferential pairing was not complete (i.e., inheritance is not fully disomic and intergeneric recombinations are observed in our study), indicated that the remaining homology is still enough to allow *Citrus* and *Poncirus* chromosome pairing, and helps understand the long-known intergeneric sexual compatibility at the diploid level (Cameron and Garber, 1968). Moreover, comparative studies of *Citrus* and *Poncirus* genetic maps revealed a high level of synteny and collinearity of markers. From unsaturated maps, Chen et al. (2008) observed only a few inversions between shared loci. Bernet et al. (2010) reported high collinearity between Fortune “mandarin” and *Poncirus trifoliata*. More recently, the availability of reference whole genome sequence allowed to compare genetic and physical maps and globally confirmed the good conservation of marker order between *P. trifoliata* and “Sunki” mandarin (Curtolo et al., 2018) and *P. trifoliata* and sweet orange (Huang et al., 2018).

Although inheritance was consistently intermediate between disomic and tetrasomic for all markers, there was considerable variation in the degree of preferential pairing. Such variation among loci is common, and has for example been observed in autotetraploid Pacific oysters (Curole and Hedgecock, 2005) and sugarcane (Jannoo et al., 2004). Variation among markers could merely reflect stochasticity, but also true differences in homology for different parts of the genome. Significant differences of the τ values were observed between LGs. LG1 displayed the higher value of random association (close to 50%) while the other LGs had a preferential disomic tendency. Disomy was very high for LG2 and LG8 with more than 85% of preferential chromosome pairing. Although an analysis of more markers would be needed to confirm the pattern, our results suggest that the divergence between “Willow leaf” mandarin and “Pomeroy” poncirus varies between chromosomes.

### Double Reduction Rates and Heterozygosity Transmission

Gametes resulting from double reduction have been detected in five loci. Double reduction implies multivalent formation and a crossover between the considered locus and its centromere with further adjacent segregation (Haynes and Douches, 1993). It results in increased homozygosity and its maximum frequency (1/6) is reached in case of systematic quadrivalent formation at meiosis (Stift et al., 2008). Our progeny sample size was not enough for accurate estimation of the double reduction rate but highlighted the possibility of tetravalent formation in the intergeneric somatic hybrid.

The diploid gametes produced by tetraploid somatic hybrids are highly heterozygous. Under tetrasomic inheritance, they should transmit either intraparental or interparental heterozygosity (Ollitrault et al., 2008), in contrast to strict disomic inheritance, which leads to exclusive transmission of interparental heterozygosity for wide interspecific somatic hybrids. When inheritance is intermediate, as found in the intergeneric *Citrus-Poncirus* Flhorag1 somatic hybrid, transmission of intraspecific and intergeneric heterozygosity depends on the degree of preferential chromosome pairing and the double reduction rates in case of tetravalent formation. As no double reduction is possible in centromeric areas, they should display higher intergeneric restitution values. Assuming preferential pairing between parental homologous chromosomes, interparental heterozygosity transmission decreases with increasing τ values. Therefore, it is logical that the interparental heterozygosity transmission (in this case intergeneric heterozygosity) observed in the intergeneric Flhorag1 somatic hybrid (90%) was much higher than the 64.1% observed for a *C. reticulata* + *C. lemon* interspecific somatic hybrid (Kamiri et al., 2011). As a consequence of this high level of intergeneric heterozygosity inheritance, the genetic diversity of the gamete population results mainly from parental intraspecific diversity segregation. However, these conclusions for genetic markers should not be extrapolated for phenotypic traits because phenotypic differentiation at the intergeneric level is very high compared with the intra-poncirus or mandarin variability.

### Implications for Breeding

Citrus breeding is hampered by complex genome structures, features of reproductive biology and the long juvenile phase, but can take advantage of vegetative propagation including apomictic seeds for rootstock multiplication, allowing clonal propagation of elite genotypes whatever its genome complexity (Ollitrault and Navarro, 2012). Therefore, breeding strategies are generally based on one (or very few) cycle of variability induction followed by direct selection of cultivars or rootstock. In such context, it is essential to optimize the transfer to the progenies, of the genetic gains obtained by phenotypic selection at the parental level. In case of *Citrus-Poncirus* intergeneric polyploid breeding, most of the genetic value comes from the combination of favorable traits from *Citrus* (tolerance to abiotic stresses such as salinity, water deficit, calcareous soils) and *Poncirus* (resistance/tolerance to diseases and pests such as tristeza virus, *Phytophthora*, nematodes; cold tolerance). It is based on dominant inheritance in highly intergeneric heterozygous structures. For further breeding using these intergeneric tetraploids as sexual parents (tetrazyg strategy; Grosser and Gmitter, 2010), it is therefore essential to transmit a large part of the parental intergeneric heterozygosity to the progenies in order to prevent overall breakage of the favorable complex multilocus genotypic structure selected at the allotetraploid parental level. From this work, it appears that the differentiation between *C. reticulata* and *Poncirus trifoliata* genomes results in preferential homologous pairing and predominantly intergeneric heterozygosity transmission. Most of the value of the somatic hybrid should thus be transmitted to its progeny. Moreover, the infrequent occurrence of non-homologous chromosome pairing offers an opportunity for intergeneric recombination, and the generation of novel allelic combinations. Several *Citrus* × *Poncirus* diploid intergeneric hybrids such as citrumello (*C. paradisi* × *P. trifoliata*), citrange (*C. sinensis* × *P. trifoliata*), and citrandarin (C. *reticulata* × *P. trifoliata*) proved their interest as rootstock and are widely used worldwide. Taking advantage of spontaneous chromosome doubling in nuclear cells, doubled diploid lines were selected for most of them (Aleza et al., 2011). If they display similar preferential disomic tendency than the “Flhorag1” somatic hybrid, they should produce gametes in which a very high proportion of the genome of the initial diploid intergeneric hybrid has been transferred. Thus, the high phenotypic value deeply selected at the diploid intergeneric parent level should have a very marked positive impact on the products of the “Tetrazyg” strategy when using these doubled-diploid parents.

## Author Contributions

PO, YF, and TK designed the experiment. DD created and provided the parental somatic hybrids the parental somatic hybrid. MK and GC performed the SSRs analysis. MK and MS analyzed the results. MK, MS, YF, and PO wrote the paper.

## Conflict of Interest Statement

The authors declare that the research was conducted in the absence of any commercial or financial relationships that could be construed as a potential conflict of interest.

## Footnotes

1 http://www.genetics.org/cgi/content/full/genetics.107.085027/DC1
