# Supplementary material for: Preferential Homologous Chromosome Pairing in a Tetraploid Intergeneric Somatic Hybrid (Citrus reticulata + Poncirus trifoliata) Revealed by Molecular Marker Inheritance
Source: Front Plant Sci. 2018 Nov 2;9:1557. doi: 10.3389/fpls.2018.01557 (PMC6224360; doi:10.3389/fpls.2018.01557)

Kamiri M., Stift M., Costantino G., Dambier D., Kabbage T., Ollitrault P. and Froelicher Y. Preferential homologous chromosome pairing in a tetraploid intergeneric somatic hybrid (Citrus reticulata + Poncirus trifoliata) revealed by molecular marker inheritance. Frontiers in Plant Science.

Supplementary Material 2: Ploidy evaluation of Chandler pummelo x (Willowleaf mandarin + Pomeroy Poncirus) progeny: mitotic chromosome counts from a young leaf preparation from a 'CHA' X 'Flhorag1' progeny sample showing 27 chromosomes counterstained with DAPI.

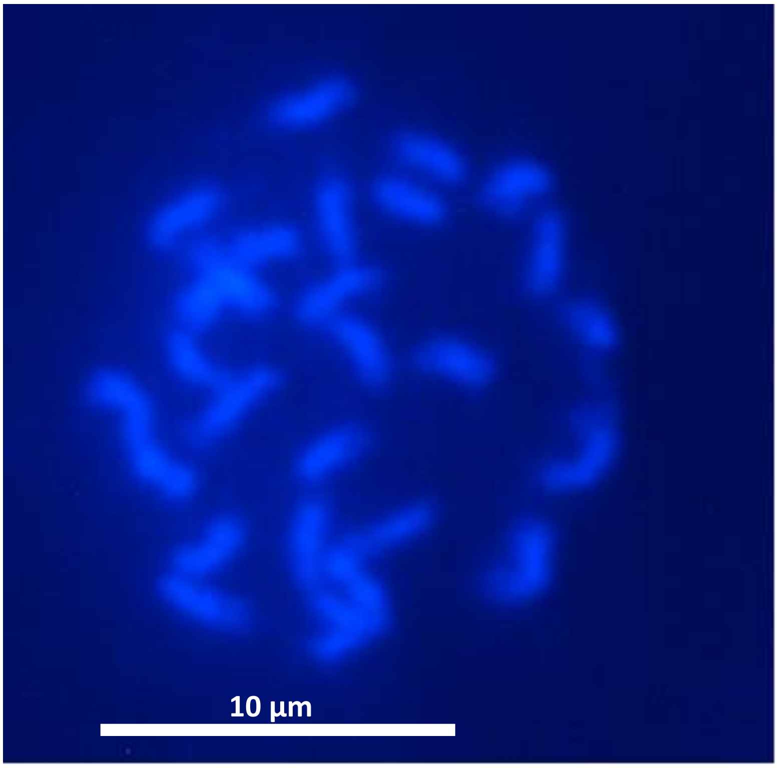

Supplement: Supplementary file 2 [file Data_Sheet_1.PDF]
