# Supplementary material for: Preferential Homologous Chromosome Pairing in a Tetraploid Intergeneric Somatic Hybrid (Citrus reticulata + Poncirus trifoliata) Revealed by Molecular Marker Inheritance
Source: Front Plant Sci. 2018 Nov 2;9:1557. doi: 10.3389/fpls.2018.01557 (PMC6224360; doi:10.3389/fpls.2018.01557)
Supplement: Supplementary file 3 [file Data_Sheet_2.PDF]

Kamiri M., Stift M., Costantino G., Dambier D., Kabbage T., Ollitrault P. and Froelicher Y. Preferential homologous chromosome pairing in a tetraploid intergeneric somatic hybrid (Citrus reticulata + Poncirus trifoliata) revealed by molecular marker inheritance. Frontiers in Plant Science.

Supplementary Material 3: Detailed segregation results, over 58 diploid gametes of 'Flhorag1' intergeneric somatic hybrid; distribution of intergeneric and intraspecific heterozygosity among 16 SSR and 9 SNP loci; average rate of thau values for each LG; rates of observed intraspecific and intergeneric recombinations by LG

LG	1	1	1	2	2	2	3	3	4	4	4	4	5	5	5	6	6	7	7	8	8	8	8	9	9			
	P1_16582061	BE5055	MEST431	P2_19903193	mCrCIR02D09	MEST46	P3_16287238	mCrCIR02G12	P4_9505439	mCrCIR02D04b	mCrCIR03D12a	mCrCIR07D06	P5_20142568	mCrCIR01F08a	mCrCIR07E12	P6_3130249	mCrCIR02F12	P7_15458045	mCrCIR07E05	P8_16631925	mCrCIR07B05	mCrCIR02G02	mCrCIR02A09	P9_12062066	mCrCIR02B07	He Intra	He Inter	Hom
1																										0%	84%	16%
2																										0%	96%	4%
3																										4%	84%	12%
4																										4%	72%	24%
5																										4%	72%	24%
6																										0%	84%	16%
7																										4%	88%	8%
8																										0%	88%	12%
9																										0%	96%	4%
10																										0%	96%	4%
11																										0%	92%	8%
12																										4%	92%	4%
13																										12%	72%	16%
14																										0%	100%	0%
15																										0%	92%	8%
16																										0%	80%	20%
17																										4%	92%	4%
18																										0%	84%	16%
19																										0%	96%	4%
20																										4%	88%	8%
21																										0%	96%	4%
22																										0%	100%	0%
23																										0%	96%	4%
24																										0%	96%	4%
25																										4%	92%	4%
26																										0%	96%	4%
27																										4%	92%	4%
28																										4%	84%	12%
29																										0%	80%	20%
30																										4%	92%	4%
31																										4%	80%	16%
32																										0%	92%	8%
33																										0%	96%	4%
34																										0%	96%	4%
35																										4%	84%	12%
36																										0%	96%	4%
37																										4%	92%	4%
38																										0%	92%	8%
39																										8%	80%	12%
40																										0%	100%	0%
41																										0%	96%	4%
42																										0%	96%	4%
43																										0%	92%	8%
44																										0%	92%	8%
45																										0%	92%	8%
46																										4%	84%	12%
47																										4%	96%	0%
48																										4%	96%	0%
49																										8%	92%	0%
50																										0%	92%	8%
51																										4%	84%	12%
52																										0%	100%	0%
53																										0%	88%	12%
54																										0%	96%	4%
55																										4%	76%	20%
56																										0%	100%	0%
57																										0%	92%	8%
58																										0%	96%	4%
He inter	86%	86%	79%	97%	95%	97%	93%	90%	90%	90%	90%	90%	90%	86%	88%	95%	93%	90%	88%	95%	95%	95%	93%	88%	83%	90.3%		
He intra	NA	5%	NA	NA	0%	NA	NA	5%	NA	2%	5%	0%	NA	NA	9%	NA	7%	NA	NA	NA	NA	5%	2%	NA	5%	4.1%		
Hom	14%	9%	21%	3%	5%	3%	7%	5%	10%	9%	5%	10%	10%	14%	3%	5%	0%	10%	12%	5%	5%	0%	5%	12%	12%			
Thau	0.42	0.41	0.58	0.11	0.07	0.10	0.21	0.21	0.31	0.31	0.23	0.31	0.31	0.41	0.36	0.16	0.21	0.31	0.36	0.16	0.16	0.16	0.14	0.42	0.38			
Average Thau /LG		0.47+/-0.09			0.09+/-0.02		0.21+/-0.00			0.29+/-0.03				0.36+/-0.05		0.18+/-0.04		0.34+/-0.03			0.15+/-0.01			0.40+/-0.02				
Intergeneric Rec.		0.29			0.09		0.14			0.24				0.26		0.10		0.16			0.14			0.26				

Intergeneric heterozygosity
Intraspecific heterozygosity
Homozyosity
